# Diabetes Distress Scale (DDS-17): European Portuguese Validation

**DOI:** 10.20945/2359-4292-2026-0035

**Published:** 2026-04-01

**Authors:** Maria Inês Serpa Paz Teixeira de Melo, Diana Falcão, Dina Isabel Campos, Mariana M. dos Santos, Jorge Hernâni-Eusébio, Anabela Barreto Silva

**Affiliations:** 1 School of Medicine, University of Minho, Braga, Portugal; 2 Family Health Unit of Minho, Braga Local Health Unit, Braga, Portugal; 3 Gualtar Family Health Unit, Braga Local Health Unit, Braga, Portugal; 4 Life and Health Sciences Research Institute (ICVS), School of Medicine, University of Minho Campus de Gualtar, Braga, Portugal; 5 Trofa Saúde Braga Sul Hospital, BRAGAFirst - Practice Based Research Network, Braga Local Health Unit, Braga, Portugal; 6 BRAGAFirst - Practice Based Research Network, Braga Local Health Unit, Braga, Portugal; 7 Sá de Miranda Family Health Unit, Braga Local Health Unit, Vila Verde, Portugal

**Keywords:** Diabetes mellitus, type 2, psychological distress, primary health care

## Abstract

**Objective:**

Diabetes mellitus (DM) is currently one of the main public health challenges, not only because of its physical complications, but also because of the emotional impact associated with its chronic management. Diabetes distress (DD) is defined as the emotional response that results from living with DM and the consequent self-care required to manage it. The Diabetes Distress Scale (DDS-17) is an instrument used to assess DD. The aim of this study was to translate and culturally adapt the DDS-17 into European Portuguese, assessing its psychometric properties.

**Subjects and methods:**

Cross-sectional study divided into three phases: translation and back-translation; cultural adaptation with pre-testing in 30 patients; and psychometric validation (descriptive analysis of the items, exploratory factor analysis and reliability analysis) with 170 patients with type 2 DM from three Primary Care Units of the Braga Local Health Unit.

**Results:**

The European Portuguese version of the DDS-17 replicated the four original factors. Internal consistency was high (total Cronbach’s alpha = 0.89), with all subscales scoring above 0.70. The overall DD was considered low (M = 1.68), but the “emotional burden related to DM” subscale showed values suggestive of moderate DD (M = 2.07).

**Conclusion:**

The European Portuguese version of the DDS-17 showed adequate validity and reliability and could be used in clinical and research contexts to better understand and manage DD in patients with type 2 DM in Portugal.

## INTRODUCTION

Diabetes mellitus (DM) has reached significant proportions worldwide and represents one of the major public health challenges in adulthood. According to the International Diabetes Federation, in 2021, 10.5% of individuals aged 20 to 79 were estimated to have DM, of whom approximately half remained unaware of their condition. Projections indicate that by 2045, one in eight adults is expected to have DM, representing a 46% increase ^(1)^. In Portugal, the estimated prevalence of DM within the same age group was 14.1% in 2021, corresponding to approximately 1.1 million individuals ^(2)^.

DM is associated with numerous physical complications, such as neuropathy, nephropathy and retinopathy, amongst others ^(3)^. However, the emotional and psychological burden linked to its chronic management has gained increasing recognition ^(4)^.

Since 2023, the American Diabetes Association has recommended a holistic approach to the management of DM, incorporating not only glycemic control and pharmacological treatment but also focus on emotional and behavioral factors. A key recommendation is the early identification of psychological conditions such as Diabetes Distress (DD), given their significant impact on patients’ quality of life and clinical outcomes ^(5)^.

DD refers to a negative emotional response associated with living with DM, including feelings of frustration, fear, overwhelm, and exhaustion related to the self-care required for disease management - such as strict glucose monitoring, treatment adherence, and concern about future complications ^(6)^. When unrecognized or inadequately addressed, DD can substantially affect treatment adherence, metabolic control, and disease progression, increasing the risk of long-term complications ^(7,8)^. Some studies have shown that DD may affect glycemic control more profoundly than major depression, representing a distinct clinical entity requiring specific assessment ^(9)^.

To address this need, psychometric tools have been developed to identify and quantify DD. The Diabetes Distress Scale (DDS-17) is one such instrument, originally created and validated in the United States by Polonsky and cols. (2005). It comprises 17 items distributed across four subscales: emotional burden (5 items); physician-related distress (4 items); regimen-related distress (5 items); and interpersonal distress (3 items). The DDS-17 demonstrated excellent internal consistency, with an overall Cronbach’s alpha of 0.93 and subscale values ranging from 0.80 to 0.95. Its use enables the accurate identification of individuals with elevated DD levels, facilitating tailored interventions and improving overall health and well-being ^(10)^.

Given the rising prevalence of DM and DD in Portugal, and the absence of validated psychometric instruments in European Portuguese, there is a clear need for a culturally adapted and validated tool to assess DD ^(2,5)^.

Currently, no specific data are available on the prevalence of diabetes distress (DD) among individuals with type 2 diabetes mellitus (T2DM) followed in primary healthcare settings in Portugal. While some studies have assessed quality of life, self care behaviors, and disease knowledge in this population, the lack of concrete data on DD reveals a major gap in national literature and underscores the need for further research ^(2)^.

Although the DDS 17 has been widely applied in other international contexts - such as Brazilian Portuguese, Mandarin, Indonesian, and Turkish - it has not yet been translated or validated for the cultural and linguistic context of European Portuguese ^(11-14)^. The absence of an adapted version represents a significant limitation for both clinical practice and research in Portugal, as it hinders the early identification of patients experiencing meaningful emotional distress.

Therefore, the present study aims to translate, culturally adapt, and validate the DDS-17 for European Portuguese, with the goal of providing a reliable, sensitive, and clinically relevant tool. This instrument will allow for a more accurate assessment of the emotional impact of DM in Portugal; enhance clinical practice by enabling early identification of patients requiring intervention for DD; and support scientific research by providing robust data on DD in the Portuguese population.

## SUBJECTS AND METHODS

This was a cross-sectional analytical study conducted in three distinct phases: translation, cultural adaptation, and psychometric validation of the DDS-17 (**Appendix 1**) for European Portuguese.

The study population consisted of patients registered in the Family Health Units (FHUs) of Minho, Gualtar and Sá de Miranda, all part of the Local Health Unit (LHU) of Braga. A convenience sample of 170 patients was obtained, selected from those seen in DM consultations at these units between March and May 2025, who met the predefined inclusion and exclusion criteria.

The sample size was determined based on methodological recommendations for validation studies of psychometric instruments, which suggest at least 100 participants and 5 to 10 participants per item ^(15)^. As the DDS-17 includes 17 items, a sample of 170 participants was chosen to ensure statistical robustness.

The inclusion criteria for participants in this study were as follows: ^(1)^ Age ≥ 18 years; ^(2)^ native European Portuguese speaker; ^(3)^ diagnosis of type 2 Diabetes Mellitus (T2DM) for more than 1 year; ^(4)^ follow-up in DM consultation at FHU of Minho, Gualtar or Sá de Miranda; and ^(5)^ under treatment with oral antidiabetic drugs and/or injectable therapies (insulin or non-insulin). Participants with ^(1)^ diagnosis of type 1 DM; ^(2)^ diagnosis of dementia or cognitive impairment; ^(3)^ severe psychiatric disorders such as bipolar disorder, schizophrenia, or psychotic disorders; and ^(4)^ conditions preventing effective verbal communication or comprehension were excluded.

### Study phases

#### Phase 1: Translation and back-translation

**Figure 1:**
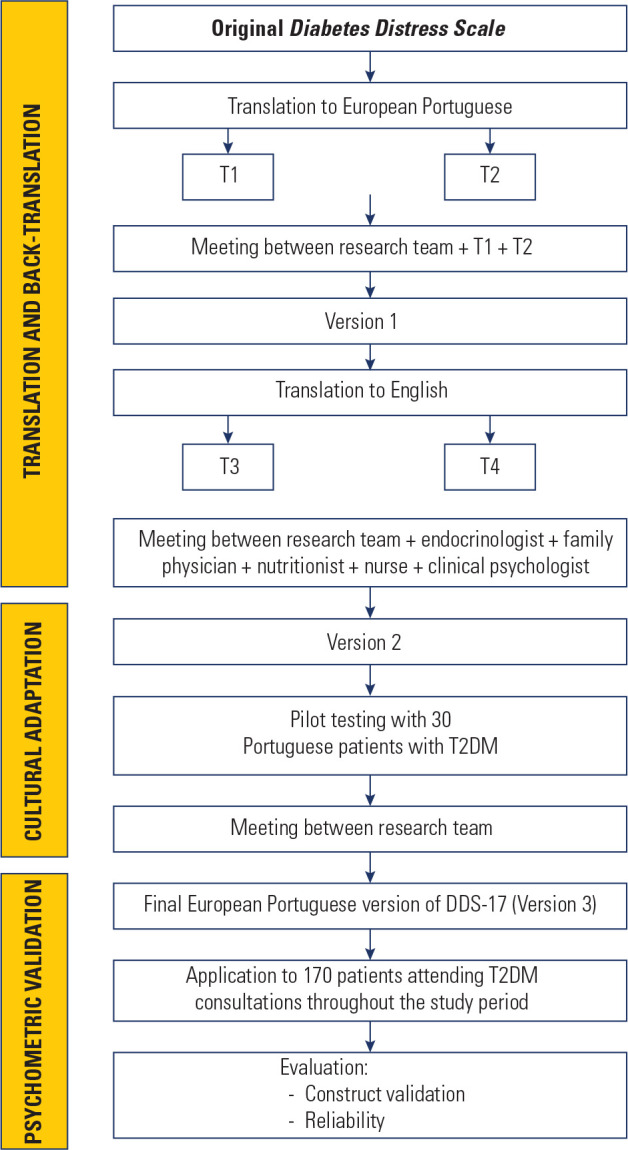
Representative flowchart of the study phases.

DDS-17: Diabetes Distress Scale; T1: Translator 1; T2: Translator 2; T3: Translator 3; T4: Translator 4; T2DM: Diabetes Mellitus type 2.

The translation phase was carried out in three stages, following specific international recommendations ^(16)^. The flowchart in **Figure 1** outlines the different phases of the study.

- **Initial translation:** The original DDS-17 (in English) was translated into European Portuguese (PT) by two independent translators (T1 and T2), both native PT speakers fluent in English.
- **Synthesis of translations:** Each translator produced a report, which was reviewed jointly by the research team and both translators to create a consensus version of the questionnaire (Version 1).
- **Back-translation:** Version 1 was then back-translated into English by two native English speakers (T3 and T4), both fluent in PT and blind to the original version. The resulting reports were compared with the original DDS-17 and analyzed for conceptual equivalence.
- **Expert panel review:** An expert panel was assembled, including an endocrinologist, a family physician with expertise in DM, a DM specialized nurse, and a clinical psychologist. Each expert received the original DDS-17, the two PT translations, Version 1, and both back-translations. A consensus meeting between the researchers and the expert panel resulted in Version 2 (pre-test version) of the scale.

#### Phase 2: Cultural adaptation

To ensure cultural adaptation, Version 2 was applied to 30 patients with T2DM followed at FHU Sá de Miranda, after confirming the inclusion and exclusion criteria. These participants did not take part in Phase 3. This phase aimed to assess the comprehension, clarity, and cultural relevance of the items. Participant feedback was compiled and reviewed by the research team, leading to the final version (Version 3), used in the next phase. All participants in Phases 2 and 3 provided written informed consent, in accordance with the principles of the Declaration of Helsinki and the Oviedo Convention.

#### Phase 3: Psychometric validation

Version 3 of the DDS-17 (**Appendix 2**) was applied to the 170 selected participants.

### Study variables

In Phases 2 and 3, sociodemographic data were collected using a structured form developed for the study. This was followed by the application of Version 3 of the DDS-17.

Authorization for the translation and validation of the DDS-17 for European Portuguese was obtained from Polonsky and cols.

### Structure of the original DDS-17

The 17 items of the DDS-17 assess DD across four subscales: emotional burden (5 items), physician distress (4 items), regimen distress (5 items) and interpersonal distress (3 items). Each item is scored using a 6-point Likert scale reflecting the frequency of emotional burden in the past month. The final score is calculated as the mean of all items and scores are interpreted as < 2.0 means little or no distress, 2.0 to 2.9 moderate distress and ≥ 3.0 high distress. Results can be analyzed as a total mean score or separately by subscale. Mean scores suggesting moderate to high distress indicate the need for clinical intervention ^(17)^.

### Statistical analysis

Data were entered and analyzed using IBM SPSS^®^ Statistics, version 29.0.2.0, 2023. Sociodemographic characteristics of the sample were described using absolute frequencies, relative frequencies, means (M), and standard deviations (SD).

Next, the psychometric properties of the Portuguese version of the DDS-17 were assessed through item-level descriptive analysis, exploratory factor analysis, and reliability analysis.

For the descriptive analysis of the 17 DDS-17 items, results were presented as mean (M), standard deviation (SD), median (Mdn), interquartile range (25^th^-75^th^ percentile), and observed range (minimum-maximum), allowing for a comprehensive assessment of DD levels within the study sample.

The factorial structure of the instrument was assessed using exploratory factor analysis with Varimax rotation, after verifying the underlying assumptions for its use - namely, a Kaiser-Meyer-Olkin (KMO) value greater than 0.50 and a statistically significant Bartlett’s test of sphericity (p-value < 0.05) ^(18)^. Given the sample size in this study, factor loadings equal or greater than 0.45 were considered acceptable ^(15)^.

Finally, scale reliability was assessed based on internal consistency. Cronbach’s alpha coefficient (α) was used, with values equal or greater than 0.70 interpreted as indicating good internal consistency ^(18)^. Additionally, corrected item-total correlation was considered as a complementary reliability criterion.

## RESULTS

A pre-test was conducted with 30 patients with T2DM using Version 3 of the DDS-17. Participants reported no difficulties in understanding the items or completing the scale, and no modifications to the translated version were deemed necessary.

According to **Table 1**, a total of 170 participants were included, recruited from three FHUs of the LHU of Braga, with a relatively balanced distribution. The sample comprised 53.5% male, with a mean age of 69.55 years (SD = 12.55), reflecting a predominantly older adult sample. Age distribution was assessed using the Kolmogorov-Smirnov (with Lilliefors correction) and Shapiro-Wilk tests, both of which indicated normality (p-value = 0.09 and p-value = 0.08, respectively) ^(15)^.

**Table 1:** Total sample characterization (n = 170)

Variable	n (%)
FHU of origin	
Minho	60 (35.3)
Gualtar	51 (30.0)
Sá de Miranda	59 (34.7)
Sex	
Male	91 (53.5)
Female	79 (46.5)
Age (years)	
Mean ± standard deviation	69.55 ± 12.55
Age range	39-96
Education (years)	
< 4	27 (15.9)
4	63 (37.1)
6-9	42 (24.7)
12	17 (10.0)
Higher education	21 (12.4)

FHU: Family Health Unit.

Educational attainment was generally low, with 53.0% of participants having completed four or fewer years of formal education, and only 12.4% having attended higher education. There were no missing data in the sociodemographic variables, and all participants completed the questionnaire in its entirety.

Table 2 summarizes the descriptive statistics for the 17 items of the DDS-17. Overall, participants provided very low scores across all items, with a median of 1.00 (“Not a problem”), indicating low levels of DD. Items 1, 7, and 12 stood out, with a median of 2.00 (“A slight problem”). Items 2, 5, 9, 11, 15, and 17 showed the least variability, with at least 75.0% of participants selecting the lowest score option on the scale (P75 = 1.00).

**Table 2:** Descriptive of DDS-17 scores about diabetes distress (n = 170)

DDS-17 question	M (SD)	Mdn (P25-75)	Min-Max
1. Feeling that diabetes is taking up too much of my mental and physical energy every day.	2.25 (1.56)	2.00 (1.00-3.00)	1-6
2. Feeling that my doctor doesn’t know enough about diabetes and diabetes care.	1.18 (0.74)	1.00 (1.00-1.00)	1-6
3. Not feeling confident in my day-to-day ability to manage diabetes.	1.56 (1.13)	1.00 (1.00-2.00)	1-6
4. Feeling angry, scared and/or depressed when I think about living with diabetes.	1.99 (1.41)	1.00 (1.00-3.00)	1-6
5. Feeling that my doctor doesn’t give me clear enough directions on how to manage my diabetes.	1.22 (0.78)	1.00 (1.00-1.00)	1-6
6. Feeling that I am not testing my blood sugars frequently enough.	1.62 (1.12)	1.00 (1.00-2.00)	1-6
7. Feeling that I will end up with serious long-term complications, no matter what I do.	2.24 (1.56)	2.00 (1.00-3.00)	1-6
8. Feeling that I am often failing with my diabetes routine.	1.82 (1.24)	1.00 (1.00-2.00)	1-6
9. Feeling that friends or family are not supportive enough of self-care efforts (e.g. planning activities that conflict with my schedule, encouraging me to eat the “wrong” foods).	1.45 (1.01)	1.00 (1.00-1.00)	1-6
10. Feeling that diabetes controls my life.	2.05 (1.46)	1.00 (1.00-3.00)	1-6
11. Feeling that my doctor doesn’t take my concerns seriously enough.	1.21 (0.81)	1.00 (1.00-1.00)	1-6
12. Feeling that I am not sticking closely enough to a good meal plan.	2.28 (1.43)	2.00 (1.00-3.00)	1-6
13. Feeling that friends or family don’t appreciate how difficult living with diabetes can be.	1.60 (1.15)	1.00 (1.00-2.00)	1-6
14. Feeling overwhelmed by the demands of living with diabetes.	1.82 (1.32)	1.00 (1.00-2.00)	1-6
15. Feeling that I don’t have a doctor who I can see regularly enough about my diabetes.	1.24 (0.84)	1.00 (1.00-1.00)	1-6
16. Not feeling motivated to keep up my diabetes self management.	1.60 (1.13)	1.00 (1.00-2.00)	1-6
17. Feeling that friends or family don’t give me the emotional support that I would like.	1.45 (1.01)	1.00 (1.00-1.00)	1-6

M: mean; Max: maximum; Mdn: median; Min: minimum; P25: 25^th^ percentile; P75: 75^th^ percentile; SD: standard deviation.

An exploratory factor analysis with Varimax rotation was conducted to examine the factorial structure of the instrument. Sampling adequacy was confirmed with a KMO > 0.50 and a significant Bartlett’s test ^(18)^. Given the sample size, factor loadings equal or greater than 0.45 were considered acceptable ^(15)^.

An initial exploratory factor analysis extracted three factors, accounting for 62.81% of the total variance. However, since four factors were expected according to the original scale, a new exploratory factor analysis was conducted, forcing a four factor solution, which explained 68.05% of the total variance (**Table 3**) ^(10)^. The first factor correspondend to the original subscale “Physician Distress”, the second to “Emotional Burden”, the third to “Regimen Distress”, and the fourth to “Interpersonal Distress”. Notably, item 3 showed a cross-loading with a relatively high loading on the first factor (0.539). However, its exclusion would result in a decrease in Cronbach’s alpha (α = 0.67) and, given its strong item-total correlation with factor 3 (0.66), it was retained in its original subscale. All items demonstrated factor loadings above 0.50.

**Table 3:** Exploratory factor analysis results

Items	1	2	3	4
2. Feeling that my doctor doesn’t know enough about diabetes and diabetes care.	0.899			
11. Feeling that my doctor doesn’t take my concerns seriously enough.	0.892			
15. Feeling that I don’t have a doctor who I can see regularly enough about my diabetes.	0.866			
5. Feeling that my doctor doesn’t give me clear enough directions on how to manage my diabetes.	0.846			
3. Not feeling confident in my day-to-day ability to manage diabetes.			0.523	
4. Feeling angry, scared and/or depressed when I think about living with diabetes.		0.809		
10. Feeling that diabetes controls my life.		0.779		
14. Feeling overwhelmed by the demands of living with diabetes.		0.761		
1. Feeling that diabetes is taking up too much of my mental and physical energy every day.		0.672		
7. Feeling that I will end up with serious long-term complications, no matter what I do.		0.652		
6. Feeling that I am not testing my blood sugars frequently enough.			0.776	
12. Feeling that I am not sticking closely enough to a good meal plan.			0.599	
16. Not feeling motivated to keep up my diabetes self management.			0.572	
8. Feeling that I am often failing with my diabetes routine.			0.566	
9. Feeling that friends or family are not supportive enough of self-care efforts (e.g. planning activities that conflict with my schedule, encouraging me to eat the “wrong” foods).				0.766
13. Feeling that friends or family don’t appreciate how difficult living with diabetescan be.				0.705
17. Feeling that friends or family don’t give me the emotional support that I would like.				0.690
% of variance explained	23.60	18.87	13.22	12.36

Table 4 presents the descriptive statistics and internal consistency results for the four DDS-17 subscales and the total score. Overall, mean scores across the subscales were low, reflecting a generally low level of DD in this sample. The “Emotional Burden related to DM” subscale showed the highest mean score (M = 2.07), exceeding the threshold of 2.0, which indicate moderate to high levels of DD that could merit clinical attention ^(17)^.

**Table 4:** Descriptive statistics and internal consistency of the DDS-17 subscales

Subscales	M (SD)	Min-Max	α
Emotional Burden related to DM	2.07 (1.13)	1.00-5.40	0.83
Physician-Related Distress	1.21 (0.74)	1.00-6.00	0.96
Interpersonal Distress	1.50 (0.89)	1.00-5.67	0.79
Regimen-Related Distress	1.78 (0.87)	1.00-4.80	0.76
Total	1.68 (0.72)	1.00-5.06	0.89

DM: diabetes mellitus; M: mean; Max: maximum; Min: minimum; SD: standard deviation; α: Cronbach’s alfa.

All subscales and the total scale demonstrated good internal consistency, with Cronbach’s alpha values above the recommended threshold of 0.70. Additionally, all items showed corrected item-total correlations above 0.30, ranging from 0.380 (item 6) to 0.924 (item 2) ^(18)^.

## DISCUSSION

The present study aimed to translate, culturally adapt, and psychometrically validate the European Portuguese version of the DDS-17, an instrument widely used to assess the emotional response associated with DM management ^(11-14)^, in a sample of 170 patients with T2DM recruited from three FHUs within the LHU of Braga.

Overall, the mean DDS-17 score was 1.68, suggesting a generally well-controlled level of DD. This may suggest effective DM management and support within the FHUs, where patients are regularly monitored and receive continuous care. Nonetheless, higher distress scores on items related to concerns about long-term complications and adherence to meal plans highlight areas that may require targeted clinical attention. These specific distress domains could affect patient self-management behaviors and ultimately glycemic control, emphasizing the need for healthcare providers to address emotional and regimen-related challenges during consultations.

The exploratory factor analysis confirmed the original four-factor structure of the DDS-17, explaining 68.05% of the total variance. The four factors - “*Physician-Related Distress*”, “*Emotional Burden*”, “*Regimen-Related Distress*”, and “*Interpersonal Distress*” - showed strong factor loadings and internal consistency, with Cronbach’s alpha ranging from 0.76 to 0.96, exceeding the recommended minimum threshold (≥ 0.70) across all subscales and for the total scale, demonstrating good reliability.

Notably, the “*Physician-related distress*” subscale presented a particularly high value (α = 0.96), exceeding that reported by Polonsky and cols. (2005), who found α = 0.88 ^(10)^. This same subscale revealed low DD levels (M = 1.21), suggesting that patients in this sample feel supported by healthcare professionals, in contrast with international studies that identify the doctor-patient relationship as a significant source of DD ^(19,20)^. This finding may be explained by the organization of Portuguese primary care services, which promote continuity and proximity between patients and physicians.

The “*Emotional burden*” subscale yielded the highest mean score (M = 2.07), reaching the threshold for moderate distress, underscoring the emotional challenges faced by individuals with T2DM, even when overall distress levels are low. This finding is consistent with previous studies that identify the emotional impact of the disease as one of the main challenges in DM self-management, particularly regarding frustration, constant worry, and feelings of overload associated with daily self-care ^(7,8)^. This result is especially relevant given the age profile of the sample, as older adults may experience additional emotional strain related to comorbidities, social isolation, or declining autonomy. Besides, highlights the importance of approaches that specifically recognize and address the psychological burden of T2DM as a chronic illness ^(21)^.

The “*Regimen-related distress*” subscale had the second-highest mean (M = 1.78), which, while not indicating clinically significant DD, may reflect a sense of monotony or fatigue associated with the daily repetition of self-care demands, even when adherence is good. These findings reinforce the need for educational approaches that foster intrinsic motivation, empowerment, and autonomy in individuals with T2DM ^(22)^.

Regarding the “*Interpersonal distress*” subscale (M = 1.50), the results suggest an overall positive perception of emotional support from family and friends. However, the low variability in responses may also reflect barriers to emotional expression, particularly among older individuals, which should be addressed in clinical practice ^(23)^.

The results indicate that the European Portuguese version of the scale demonstrates robust psychometric indicators of validity and reliability, making it suitable for clinical and academic use in Portugal. In line with the original structure of the scale, the present findings confirmed that the distribution of items across the four proposed subscales is appropriate for the Portuguese context. Studies conducted in Brazil, China, Turkey, and Indonesia have also yielded similar structures, reinforcing the relevance of the subscale domains and confirming the psychometric stability of the DDS-17 across diverse settings, regardless of cultural differences or healthcare system characteristics ^(11-14)^.

Despite promising results, this study presents limitations that should be considered. The sample was one of convenience and geographically restricted to the Minho region, which may limit the generalizability of findings to other populations. The study sample was characterized predominantly by older adults (mean age 69.55 ± 12.55 years) with relatively low educational attainment, characteristics that may influence how DD is experienced and reported. Although this age group represents a substantial proportion of the Portuguese population with T2DM, it limits broader applicability ^(2)^. Reduced health literacy may have affected item comprehension or subjective interpretation, potentially leading to underreporting of emotional distress. Another key limitation is the absence of temporal stability testing (test-retest reliability) and sensitivity to change over time which are critical aspects for using the DDS-17 in longitudinal clinical monitoring or intervention evaluation.

Nonetheless, this study makes a relevant contribution to clinical practice in Portugal by providing a psychometrically validated tool to assess DD. Its regular use may support early detection of clinically significant emotional distress, enabling targeted and personalized interventions, in alignment with American Diabetes Association guidelines for comprehensive DM care. Future studies should extend the application of the DDS-17 to more diverse populations and examine its association with other relevant variables such as treatment adherence, disease duration, presence of complications, and mental health indicators. Evaluating concurrent validity with instruments such as the PHQ-9 or HADS could further strengthen the diagnostic utility of the European Portuguese version of the DDS-17. Additionally, assessment of its temporal stability would help confirm its applicability in longitudinal research and clinical follow-up contexts.

In conclusion, the validation of the DDS-17 for European Portuguese represents a valuable contribution to clinical practice in Portugal, addressing the lack of culturally adapted instruments for assessing DD. Based on its strong internal consistency, robust factorial structure, and ease of use, the DDS-17 proves to be suitable for application in the Portuguese context, particularly within Primary Health Care, allowing for the early detection of emotional dimensions that are often overlooked in the management of T2DM. The systematic use of the DDS-17 may support a biopsychosocial, person-centered approach to T2DM care, facilitating the early identification of DD and guiding personalized interventions that complement metabolic control. Future studies are needed to explore associations with other variables and to assess the temporal stability of the DDS-17.

## Data Availability

datasets related to this article will be avail-able upon request to the corresponding author.

## Appendix

**Appendix 1:** Diabetes Distress Scale

	Not a Problem	A Slight Problem	A Moderate Problem	Somewhat Serious Problem	A Serious Problem	A Very Serious Problem
1. Feeling the diabetes is taking up too much of my mental and physical energy every day.	1	2	3	4	5	6
2. Feeling that my doctor doesn’t know enough about diabetes and diabetes care.	1	2	3	4	5	6
3. Not feeling confident in my day-to-day ability to manage diabetes.	1	2	3	4	5	6
4. Feeling angry, scared and/or depressed when I think about living with diabetes.	1	2	3	4	5	6
5. Feeling that my doctor doesn’t give me clear enough directions on how to manage my diabetes.	1	2	3	4	5	6
6. Feeling that I am not testing my blood sugars frequently enough.	1	2	3	4	5	6
7. Feeling that I will end up with serious long-term complications, no matter what I do.	1	2	3	4	5	6
8. Feeling that I am often failing with my diabetes routine.	1	2	3	4	5	6
9. Feeling that friends or family are not supportive enough of self-care efforts (e.g. planning activities that conflict with my schedule, encouraging me to eat the “wrong” foods).	1	2	3	4	5	6
10. Feeling that diabetes controls my life.	1	2	3	4	5	6
11. Feeling that my doctor doesn’t take my concerns seriously enough.	1	2	3	4	5	6
12. Feeling that I am not sticking closely enough to a good meal plan.	1	2	3	4	5	6
13. Feeling that friends or family don’t appreciate how difficult living with diabetes can be.	1	2	3	4	5	6
14. Feeling overwhelmed by the demands of living with diabetes.	1	2	3	4	5	6
15. Feeling that I don’t have a doctor, who I can see regularly enough about my diabetes.	1	2	3	4	5	6
16. Not feeling motivated to keep up my diabetes self-management.	1	2	3	4	5	6
17. Feeling that friends or family don’t give me the emotional support that I would like.	1	2	3	4	5	6

Polonsky WH, Fisher L, Earles J, Dudl RJ, Lees J, Mullan J, et al. Assessing Psychosocial Distress in Diabetes Development of the Diabetes Distress Scale. 2005.

**Appendix 2:** Diabetes Distress Scale

	Não é um problema	É um problema ligeiro	É um problema moderado	É um problema algo sério	É um problema sério	É um problema muito sério
1. Sinto que a diabetes está a tirar-me muito da minha energia mental e física todos os dias.	1	2	3	4	5	6
2. Sinto que o meu médico não sabe o suficiente sobre a diabetes e os cuidados de saúde relativos à diabetes.	1	2	3	4	5	6
3. Não me sinto confiante com a minha capacidade de gerir a diabetes no meu dia-a-dia.	1	2	3	4	5	6
4. Sinto-me zangado(a), assutado(a) e/ou deprimido(a) quando penso em viver com diabetes.	1	2	3	4	5	6
5. Sinto que o meu médico não me dá orientações claras o suficiente sobre como gerir a minha diabetes.	1	2	3	4	5	6
6. Sinto que não estou a testar os meus níveis de açúcar no sangue com a regularidade suficiente.	1	2	3	4	5	6
7. Sinto que vou acabar por ter complicações sérias a longo-prazo, independentemente do que faça.	1	2	3	4	5	6
8. Sinto que falho frequentemente com a minha rotina da diabetes.	1	2	3	4	5	6
9. Sinto que os meus amigos ou família não me apoiam o suficiente nos meus esforços de autocuidado (por exemplo: planeiam atividades que entram em conflito com o meu horário, incentivam-me a comer os alimentos “errados”).	1	2	3	4	5	6
10. Sinto que a diabetes controla a minha vida.	1	2	3	4	5	6
11. Sinto que o meu médico não leva em conta as minhas preocupações de forma séria o suficiente.	1	2	3	4	5	6
12. Sinto que não estou a cumprir adequadamente um bom plano alimentar.	1	2	3	4	5	6
13. Sinto que amigos ou família não valorizam o quão difícil pode ser viver com diabetes.	1	2	3	4	5	6
14. Sinto-me sobrecarregado(a) com as exigências de viver com diabetes.	1	2	3	4	5	6
15. Sinto que não tenho um médico a quem possa recorrer com a regularidade suficiente acerca da minha diabetes.	1	2	3	4	5	6
16. Não me sinto motivado(a) para manter a autogestão da minha diabetes.	1	2	3	4	5	6
17. Sinto que amigos ou família não me dão o apoio emocional que gostaria.	1	2	3	4	5	6

Polonsky WH, Fisher L, Earles J, Dudl RJ, Lees J, Mullan J, et al. Assessing Psychosocial Distress in Diabetes Development of the Diabetes Distress Scale. 2005.
